# Book Reviews

**Published:** 1832-09

**Authors:** 


### BIBLIOGRAPHICAL NOTICE.

*The Anatomy and Physiology of the Organ of Hearing; with Remarks on Congenital Deafness, the Diseases of the Ear, some Imperfections of the Organ of Speech, and the proper Treatment of these several Affections.* By David Tod, Member of the Royal College of Surgeons.—8vo. pp. 147. Longman and Co., London.

We knew little of the physiology of hearing (properly so called,) before we opened this volume, and we still know but little now that we have closed it. Not that Mr. Tod has merely written "a book," being anxious to see his name in print, and knowing that "a book is a book, although there's nothing in't;" but that the subject is one at present involved in so much obscurity, and the collateral sciences which should aid in the solution of the problem are in so rudimentary a state, that we doubt whether a rational physiology of the organ of hearing is an achievement that will reward the exertions of the present age.

We must, we are afraid, be content to act as pioneers, and open the road for our successors, in the same manner as our predecessors have laboured for us, and left the materials whence, in other points, many a fair discovery has been made. The science of sound has, indeed, advanced more within the last few years than it had done for the previous century; and the discoveries of Chladric, Willis, Farraday, and Wheatstone, will not, we are assured, be without their effect in assisting the physiologist to explicate the functions of the several parts of the organ of hearing, and to trace the series of causes and effects through this most beautiful piece of mechanism with something like philosophical perspicuity. But acoustics alone will not suffice: before the functions of the ear can be hoped to be explained, the structure must be correctly known, and here Mr. Tod has "done the state some service;" he has, with much assiduity and patience, dissected the different parts minutely, and has distinguished several muscular fasciculi not hitherto specifically described, and which, in such an organ, are probably of as much importance as larger contractors and flexors in larger parts. The plates and the descriptions of internal structure are good, and, on the whole, the anatomical sections extremely creditable. We recommend their perusal to students in general.

The so-called "physiology of the ear" is far less satisfactory; but for this we impute not blame to the author. We have rarely read a book, or listened to a lecture, on hearing, however anxious we might be, and have been, to gain information on the subject, that did not seem "verba non amplius:" still there are here, amongst much crude hypothesis, two or three interesting experiments and cases recorded, from which we shall make our extracts, rather than from the more speculative parts of the essay.

"*Experiment* 1. I took a silver tube, and introduced it through the meatus of one of my ears, until I felt it touch the membrana tympani, having plugged the other meatus with the point of one of my fingers. I then began to listen. The first thing perceived was the total absence of the power of distinguishing the distance of sounds, and the quarter from whence they came; and the second was the pain which they produced, apparently from their harshness.

"*Experiment* 2. I took a cat, and cut the auricle of one ear completely out, and brought the margins of the skin together by suture, so as to obliterate the orifice of the meatus externus. After the animal recovered, nothing of any importance could be discovered in her œconomy for four or five weeks; but after that period she began to exhibit, occasionally, a considerable degree of alarm, and to run up and down stairs several times a day, with great rapidity, until she became much exhausted. With these paroxysms of terror she was visited for nearly a month, when they entirely left her. Considering her symptoms to be very strange, I frequently watched her motions, in order to learn, if possible, the cause. When she awoke from sleep, I observed that she appeared as if confused and alarmed by the passing sounds; for she almost invariably started up suddenly, then by moving her remaining auricle endeavoured to ascertain the direction of the sounds which appeared to disturb her, and afterwards ran off in great agitation. These symptoms, however, soon began to assume a milder character, and at last they entirely left her. After she got well, I clearly perceived that she could not discover the quarter from whence a sound came, with that facility which cats are known to possess; for she always moved her head, and also her auricle, in different directions, before she appeared aware of the quarter from whence any slight sound emanated.

"From what we have now stated, there cannot, I think, exist a doubt as to the auricle having been provided for the purpose of enabling animals to discover the accidental or local properties of sounds. But there are other offices which the auricle must also perform; for if everything is, as we have reason to believe, of a compound nature, and has a form coinciding with its properties, it is very obvious that the irregular concave surfaces of the auricle must endow it with the power of collecting all the sonorous properties, as well as of responding to those which we call local or accidental. Hence, among those animals which make extensive use of their auriculæ, we notice that they invariably turn their concave surfaces towards the sonorous body, and keep them fixed in that position as long as they are listening attentively: further, that they have the power of varying the figure of the auricular surfaces according to circumstances. But the human auricle has, as already stated, very little motion, and therefore power of accommodating itself to these circumstances; for although it has an irregular concave surface leading to the meatus externus, its muscles have very limited powers over its motions. For this apparent defect, Nature has provided a substitute of a less complex, and of a more ornamental description; for, when it is requisite to listen attentively to distant or obscure sounds, we instinctively open our mouth, and by this simple process enlarge the orifice of the meatus externus, through the medium of the condyloid processes of the lower jaw, which, from forming an imperfect or compound ginglymoid joint, draws the adjacent soft parts forwards and downwards, and thereby enable more sonorous properties to be conveyed to the surface of the membrana tympani." (P. 42.)

The author's observations on the treatment of congenital deafness, we fear, are more physiologically curious than likely to become of practical importance. Nevertheless, we grant that the principle of exciting parts which may be congenitally deficient or only partially developed, is one that has reason on its side, and deserves to be extensively tried: a few cases of failure should not lead to despair, and although even the best of those recorded by the author can scarcely be called successful, still there seems to have been sufficient improvement to encourage a repetition of the treatment; for, as he observes,

"We have reason to think, from the spontaneous cures recorded to have taken place at particular stages of life, that the vital principle in those parts is susceptible of peculiar excitement, and that if it does not generate structures hitherto defective, it can, at least, produce such a revolution in the textures that exist, as to make them subservient to the original intentions of their formation. Ought we not then to endeavour, by every means in our power, to solicit such fortunate issues?" (P. 105.)

As a case somewhat in point, although not relating to the developement of the structures of the internal ear, we can add an instance of parts congenitally latent, the evolution of which was casually excited by local irritation: it occurred in a gentleman who remained an *imberbis* long past the usual age, and who, chancing to have a boil form on the left cheek, found subsequently that a whisker grew on that side of the face, while the other cheek remained as barren as before.

We extract the most successful of the cases reported.

"Case I. Sarah Fulford, ætat. ten, of No. 8., Great Swan alley, Coleman street, a girl of a good but delicate constitution, and apparently free from all hereditary complaints. At birth she was observed to cry very distinctly, and afterwards, when asleep, no noise could awake her. Little attention was paid to these circumstances until she was nearly two years old, when she was discovered to be deaf and dumb, and in that state she has continued ever since. To-day, March 10, 1828, she was placed under my care. I learned that she could hear the sound of a bell when rung close to her, and that with great difficulty she could articulate several words, such as father, mother, beer, &c., which she had learnt from the motions of the lips of others.

"On examining the meatus of each ear, there appeared to be a plentiful secretion of wax, and the membrana tympani seemed to be entire, but apparently more concave than usual. On introducing the soft bougie, however, I found that although the membrane appeared perfect to the eye, it was very much malformed; that the handle of the malleus was external to it, and attached to the incus instead of the malleus. The right ear was still more malformed, and more insensible to sound than the left. Considering the case to be capable of relief, I explained to the parents the importance of her living in a pure air, and having her digestive organs strictly attended to. Ordered a perpetual blister to the nape of the neck, and a mild aperient pill to be taken every night.

"March 17. Hearing and speech a little improved: syringed both ears with tepid water, and plugged them with lint moistened in linimentum camphoræ. Ordered the former remedies to be continued.

"March 24. Hearing and speech continuing to improve; and she now notices and articulates the simpler sounds with facility. Was told that, since she began to improve, she has evinced a great desire to learn to read, and that she can now repeat many of the letters of the alphabet. Ordered all the remedies to be continued.

"March 31. Speech greatly improved; can now ask for various articles, and execute errands with some facility. Hearing also improved, but apparently not so much as the speech. General health considerably better. Ordered all the remedies to be continued.

"April 14. Hearing and speech continuing to improve; can now hear what is said, but is unable to make a reply, evidently shewing the speech to be in its infancy. Was told that she pays more attention to the conversation of children than adults. Applied a few drops of æther rectificatus to each tympanum, which excited much pain. Ordered all the former remedies to be continued.

"April 20. Hearing and speech progressively improving; and I was told to-day that she had heard the music of an organ in the street, apparently for the first time, which delighted her much. Syringed each ear, and brought away a quantity of adventitious membrane. Ordered all the remedies to be continued.

"April 27. Hearing and speech considerably improved; can hear better with the right ear than the left. Removed from the former, by the syringe, a quantity of membrane. Ordered all the remedies to be continued.

"May 11. Hearing and speech improving. Omitted the æther, and applied a little tinct. cantharidis instead. Ordered the other remedies to be continued.

"May 18. Continuing to improve. Ordered the blister to be healed.

"May 22. Continuing to improve.

"At every visit after this period she displayed a progressive improvement n her hearing, speech, and general health; and in a short time she appeared to require nothing more than a teacher.

"N.B. Sometime after this patient ceased her attendance on me, her parents, from being poor and having a family of young children, obtained an admission for her into the Deaf and Dumb Asylum, Kent road, although she was not a very fit object for the institution. There she remained for some time, but was subsequently sent home, and died of pneumonia. From not hearing of her death till some months after it had occurred, I had no opportunity of examining the state of the ears by dissection." (P. 109.)

The following is a very interesting case, and analogous in some respects to one recorded in vol. liv. p. 519, of our Journal, and referred to by the author.

"It has been stated, that during the infantile period of life the osseous textures of the ear are among the softest in the body, and that they appear from this circumstance to be very liable to disease, particularly in scrofulous subjects. A few weeks ago, my friend Mr. Linnecar brought me a piece of bone, including the entire labyrinth, which had come away, by the meatus externus of the left ear, from a patient of his, whom he invited me to visit in company with himself. On examining the bone, it appeared that the whole of the petrous portion had, like the shaft of a tibia when affected with necrosis, lost its vitality, and been detached by the ordinary process of nature. As the case was the first of the kind I had ever seen or heard of, and one which would probably be the means of establishing some of the opinions I had long entertained, (and which have been noticed in this treatise,) I felt great anxiety to have the circumstances attending it investigated; and in a short time I had the patient before me, a fine lively girl, about two years and a half old, but apparently predisposed to scrofula. On examining the child, nothing very particular could be discovered. Matter of a healthy appearance was exuding through the meatus externus of the left ear; the face on the same side was a little swollen, and had lost its powers of motion, but not of sensation; for she could not bear the prick of a pin without crying. Her general health was apparently good. From Mr. L. I learnt that he had attended a sister of the patient for diseased mesenteric glands, but that she had not exhibited any symptom of a scrofulous nature; that about twenty months ago (April 1830) he was desired to see the child, then about eight months old, and found her labouring under symptoms of phrenitis, which he treated in the usual way, and the patient soon recovered. In about a month or six weeks after this attack, a purulent discharge was observed to flow from the left ear (the child at the time being in apparent good health); but no notice was taken by the parents of this circumstance until after the expiration of three or four weeks, when the mother, on observing the face and neighbouring parts looking red, became alarmed, and sent for Mr. Linnecar again. On examining the ear, he now found the discharge to be copious, of a purulent character, and excoriating the parts over which it passed: there was no complaint of pain, no heat or swelling about the ear; but the left side of the face, including the eye, seemed larger than the right, and the left eye was never closed during sleep. The patient seemed in good general health, for she ate, drank, and slept well, and was beginning to walk. Various remedies were applied; but the symptoms continued much the same, with the exception of the discharge, which had increased. At length the left side of the face became perfectly paralysed, and shortly afterwards the ossicula auditus were discharged through the meatus externus. Mr. L. then lost sight of the child for three months, in consequence of her being removed into the country. On her return, he found her labouring under irritative fever and disordered digestive organs, although the child in the interval had been apparently in good health. The discharge still continued from the ear, accompanied with a most offensive odour. A fungous excrescence was now observed filling the meatus, whilst the auricle was pushed out from the side of the head; and the child cried when the finger was applied to the posterior part of the organ, although there was no sign of inflammation. On applying the cupri sulphas to the fungus, it came away, but still continued growing rapidly, and required the escharotic to be frequently repeated: each time it was removed he perceived a piece of bone gradually advancing from the bottom of the cavity. This at length came away spontaneously, and on examination proved to be the entire petrous portion of the temporal bone. Upon looking into the ear, Mr. L. could now see a considerable distance down the meatus, which appeared at the bottom to be lined with healthy-looking pus. Since then there has been scarcely any discharge. The external ear has been gradually returning to its natural size, and no one would now suppose so serious a disease had ever existed."* (P. 134.)

---

*Clinical Reports of the Surgical Practice of the Glasgow Royal Infirmary.* By John Macfarlane, m.d., Member of the Faculty of Physicians and Surgeons of Glasgow; senior Surgeon to the Royal Infirmary; Lecturer on Clinical Surgery, &c.—8vo. pp. 314. David Robertson, Glasgow; Adam Black, Edinburgh; and S. Highley, London.

This is a very sensible and well-written volume, and a valuable addition to our stores of clinical information: it is replete with practical interest, containing well-digested and sufficiently copious reports of upwards of a *hundred and fifty* cases, including some of the most important diseases that fall under the care of the surgeon; such as aneurism of various kinds, 9 cases; tumors, adipose, sarcomatous, carcinomatous, &c. 26 cases; herniæ, 9 cases; vesical calculus, 15 cases; wounds and diseases of the pelvic viscera, 17 cases; with fractures, dislocations, diseases of the joints and of the spine; burns, tetanus, lupus, &c., thus forming, on the whole, as varied a collection as the most wayward could desire, and at the same time as philosophic a common place book as the most fastidious could demand. We regret that our limits will not allow us to make any extracts now, but we shall occasionally select some of the most instructive cases for our Hospital Reports. In the meantime we recommend its perusal most cordially to our readers.

---

